# Why Some Patients Undergoing Lipoprotein Apheresis Therapy Develop New Cardiovascular Events?

**DOI:** 10.3390/jcdd7030025

**Published:** 2020-07-16

**Authors:** Ulrich Julius, Solveig Kuss, Sergey Tselmin, Ulrike Schatz, Stefan R. Bornstein

**Affiliations:** Lipidology and Center for Extracorporeal Treatment, Department of Internal Medicine III, University Hospital Carl Gustav Carus at the Technische Universität Dresden, Fetscherstr. 74, 01307 Dresden, Germany; solveig_frieda_rosa.kuss@mailbox.tu-dresden.de (S.K.); sergey.tselmin@uniklinikum-dresden.de (S.T.); ulrike.schatz@uniklinikum-dresden.de (U.S.); stefan.bornstein@uniklinikum-dresden.de (S.R.B.)

**Keywords:** lipoprotein apheresis, cardiovascular events, LDL-cholesterol, LDL-cholesterol corrected for its content in Lipoprotein(a) particles, Lipoprotein(a), targets for LDL-cholesterol

## Abstract

Lipoprotein apheresis (LA) is an effective tool to reduce cardiovascular events (CVEs) in high-risk patients with elevations of low density lipoprotein-cholesterol (LDL-C) and/or Lipoprotein(a) (Lp(a)). All patients included into this retrospective analysis had experienced CVEs before the start of the LA therapy. We compared personal and lab data in two groups: CVEx/0 (*n* 60) with no new events during LA therapy, CVEx/1+ (*n* 48) with at least one new event. Patients of Group CVEx/1+ were about 5 years older when they had started the extracorporeal therapy, and they experienced more CVEs prior to that timepoint. There was a positive correlation between the number of CVEs before and during LA therapy. No differences were seen with respect to lipid concentrations, even after a correction of LDL-C concentrations for the LDL-C transported with Lp(a) particles. LA sessions effectively reduced both LDL-C and Lp(a). Lp(a) levels measured before LA sessions were lower than those measured initially. It appeared difficult to reach the target values for LDL-C published in the ESC/EAS Guideline in 2019, although all patients were maximally treated including drugs when tolerated. In conclusion, it will be important to initiate an LA therapy earlier, at least after a second CVE and at a younger age.

## 1. Introduction

In high-risk patients lipoprotein apheresis (LA) is the last resort therapy to prevent new cardiovascular events (CVEs) [1]. In two studies—one with a retrospective evaluation and one with a prospective design—the high effectiveness of LA with respect to a reduction of CVEs by more than 80% was shown when comparing the situation before the start of the extracorporeal therapy and during this therapy in patients with elevated lipoprotein(a) (Lp(a)) levels [2,3,4]. In the last years, the number of patients with this indication was constantly increasing in Germany. In patients who show only high LDL-cholesterol (LDL-C) concentrations and whose Lp(a) was absent or in the normal range the efficiency of LA with respect to CVEs is less [5].

When the Federal Joint Committee (Gemeinsamer Bundesausschuss) published its decision to accept an LA in patients with high Lp(a) levels, a randomized controlled study was requested [6]. However, the ethics committee did not allow this kind of study to be performed. Thus, the usual way to compare outcome data in LA patients is to look at the situation before and during the extracorporeal therapy.

The aim of this evaluation was to look for factors that may be associated with the incidence of new CVEs in LA patients. In general, both personal factors and standard laboratory data have been taken into consideration. A special focus was made on the concentrations of LDL-C after a correction for the LDL-C which is contained in the Lp(a) particles.

About 10 years ago, we asked the same question as we are now [7]. However, since then the number of patients at our facility has more than doubled; all new patients starting the LA therapy in the last years had an elevation of Lp(a) and the quality of the measurement of this parameter was improved.

## 2. Material and Methods

At the timepoint of this evaluation (May 2019) a total of 132 patients were treated with LA at our department. Four patients were not included because two of them had undergone a heart transplantation and the other two did not suffer from CVEs (but had significant atherosclerotic lesions at several vessel regions and belonged to families where relatives had CVEs). Moreover, in order to ensure an observation time of at least more than 1 year, during the LA treatment, patients who started LA in 2018 were also not taken into consideration (*n* 20). The remaining 108 patients were subdivided into two groups: 1. CVEx/0 patients reported events before the start of LA and had no events during LA (*n* 60), 2. CVEx/1+ patients reported events before the start of LA and suffered from at least one CVE at the follow-up during LA (*n* 48). The following CVEs were taken into account: myocardial infarction, stroke, transitory ischemic attack, occlusions of arteries, interventions (stents, operations) at various vessel territories. The majority of new events during LA were stenting.

Lipid concentrations (triglycerides (TG), high density lipoprotein-cholesterol (HDL-C), LDL-cholesterol (LDL-C)) have been measured using routine methods (Cobas8000-System from Roche, Basel, Switzerland.). For Lp(a) measurement the Immunoassay for Lp(a) (WHO/IFCC International Reference Reagent) – SRM 2B (Roche/Hitachi Cobas c 701/702) was used.

The directly measured LDL-C reflects two compartments: 1. Transported with the LDL particles, 2. Transported with the Lp(a) particles. Drugs decreasing LDL-C concentrations exert an effect on the LDL-C compartment which is contained in the LDL particles. In order to get an information about this compartment we subtracted the LDL-C in Lp(a) from the measured LDL-C. Two steps were needed for this calculation: 1. Lp(a) levels which are given in nmol/L had to be converted into mg/dL (by dividing by 2.4)—data show that this calculation is evidently not correct, 2. We estimated the LDL-C content in the Lp(a) particles to be equivalent to 30%—which is an accepted estimation. The formula used was: LDL-Ccorrected (LDL-Ccorr) = LDL-C - 0:3 × Lp(a) mass [8]. Thus, the so-called LDL-Ccorr concentrations may not be exact but they provide an idea about the compartment which can be optimized by drugs.

In order to describe the lipid load in the patients during LA therapy, interval mean values have been calculated for LDL-C according to Kroon [9] and for Lp(a) according to Tselmin [10].

At our department, 6 different LA methods have been used (Table 1) [11].

Up to now, no clear indication for a certain LA method exists. Thus, patients started treatment with a given LA method for which a free machine was available. In some patients, the LA method was switched to another one when the effectiveness with respect to lipid lowering rates was not sufficient or when adverse effects occurred. In this study, this switching was not documented. The vast majority of our patients were treated with LA weekly.

We report lipid concentrations measured before the first LA (the lipid levels after the first LA session are not representative, because usually at this session the treated whole blood or plasma volume has to be reduced in order to ensure a high safety level for the patient) and LDL-C and Lp(a) levels observed before and after a single LA session performed in May 2019.

The majority of our patients were taking lipid-lowering drugs (statins when tolerated, ezetimib, PCSK9 inhibitors in some patients). Usually this therapy had been already started following CVEs (mostly several months before the initiation of the LA therapy) and was modified in those patients whose LDL-C values appeared to be rather high during the extracorporeal therapy.

The numbers of lipid-lowering drugs were in the groups: 1. CVEx/0 at the start of LA therapy: statins 54 (90%), ezemitib 28 (46.7%), PCSK9 inhibitors 0, in May 2019: statins 46 (76.6%), ezetimib 27 (45%), PCSK9 inhibitors 9 (15%); 2. CVEx/1+ at the start of LA therapy: 42 (87.5%), ezetimib 23(47.9%), PCSK9 inhibitors 2 (4.2%), in May 2019: statins 38 (79.2%), ezetimib 24 (50%), PCSK9 inhibitors 13 (27.1%).

For comparisons of numerical data, the one-way ANOVA was used (IBM SPSS Statistics Version 25.0, Armonk, NY, USA) with post-hoc test according to Bonferroni. A correlation analysis according to Pearson was performed in Group CVEx/1x between CVEs before the start of LA therapy and CVEs during LA therapy. A p-level below 0.05 was considered to be significant.

The study had been approved by the local ethics committee (EK 82022019).

## 3. Results

### 3.1. Groups Defined by CVEs during LA Treatment

Group CVEx/0 consisted of 60 patients (42 males, 18 females), the mean age at the first LA session was 55 years (range 29–75 years). In the mean, patients of this group were treated with LA for 5.1 years (range 1.4–21.8 years. In total, 48 patients (28 males, 20 females) belonged to group CVEx/1+, their mean age at first LA session was 60 years (range 41–75 years), and the LA treatment was performed for 6.3 years in the mean (range 1.4–26.4 years).

The age difference between both groups was statistically significant (*p* 0.041). The gender distribution and the duration of the LA therapy were not different.

In the total population, before LA therapy 2.71 CVEs occurred per patient in the mean, during the mean follow-up time of more than 5 years during LA this number amounted to 1.06 CVEs per patient. This means a relative reduction of CVEs by about 61%. Nevertheless, the majority of our patients did not develop new CVEs during the extracorporeal therapy (Group CVEx/0).

**Figure 1:**
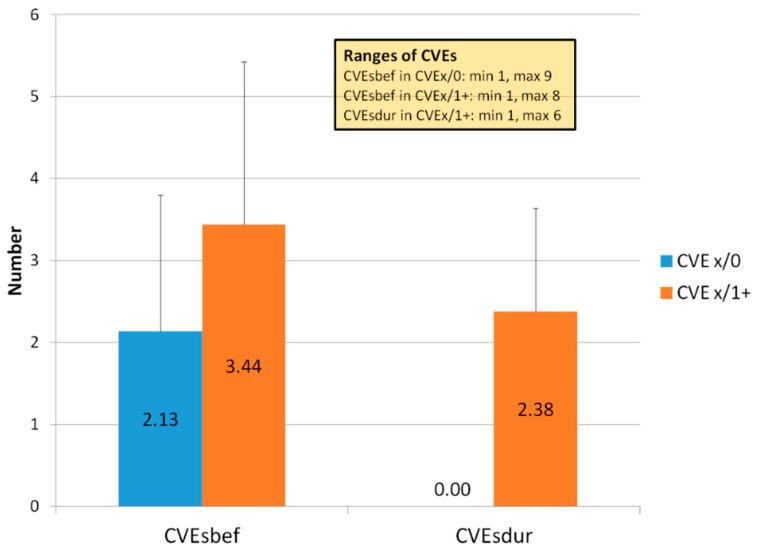
Number of CVEs per patient (means, standard deviations) before the start of LA therapy (CVEsbef) and during LA therapy (CVEsdur) in both groups.

The mean numbers of CVEs per patient in both groups are given in Figure 1.

The number of CVEs before the start of the LA therapy was statistically significantly different (*p* 0.002) between the two groups. Moreover, in the Group CVEx/1+ the number of CVEs before LA therapy positively correlated with the CVEs number during LA treatment (correlation coefficient 0.331, *p* 0.022).

### 3.2. Lipid Concentrations in the CVE-Groups

**Figure 2:**
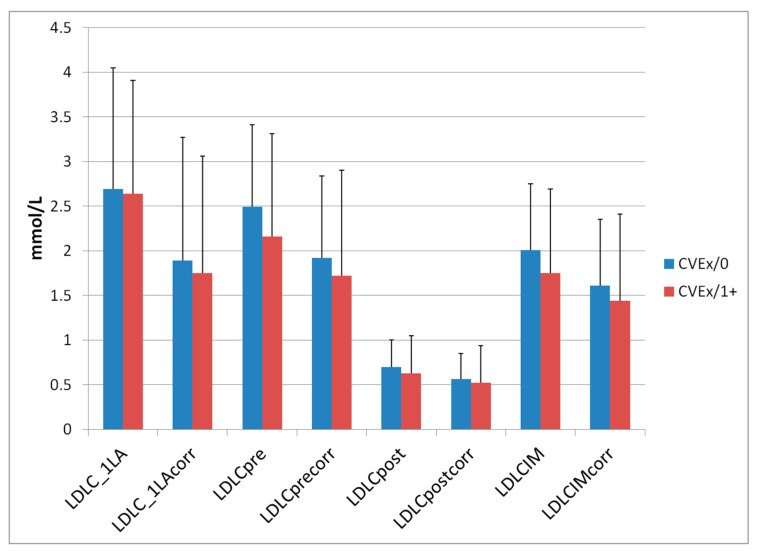
LDL-C and corresponding LDL-Ccorr levels (means, standard deviations) at first LA session and at an LA session in 2019 (before (pre) and after (post) one single LA session).

Figure 2 depicts the LDL-C concentrations measured before the first LA session (reflecting starting conditions), LDL-C levels before and after a single LA session in May 2019, interval mean values and the corresponding LDL-Ccorr concentrations (which were corrected for the LDL-C contained in the Lp(a) particles at the given timepoint).

Initial LDL-C concentrations ranged in Group CVEx/0 between 0.38 and 7.20 mmol/L, before the LA session in 2019 between 0.53 und 4.95 mmol/L and after this session between 0.14 and 1.60 mmol/L. In Group CVEx/1+, the initial LDL-C was between 0.62 and 6.11 mmol/L, before the LA session in 2019 between 0.59 and 5.91 mmol/L and after the LA session between 0.13 and 2.56 mmol/L.

**Figure 3:**
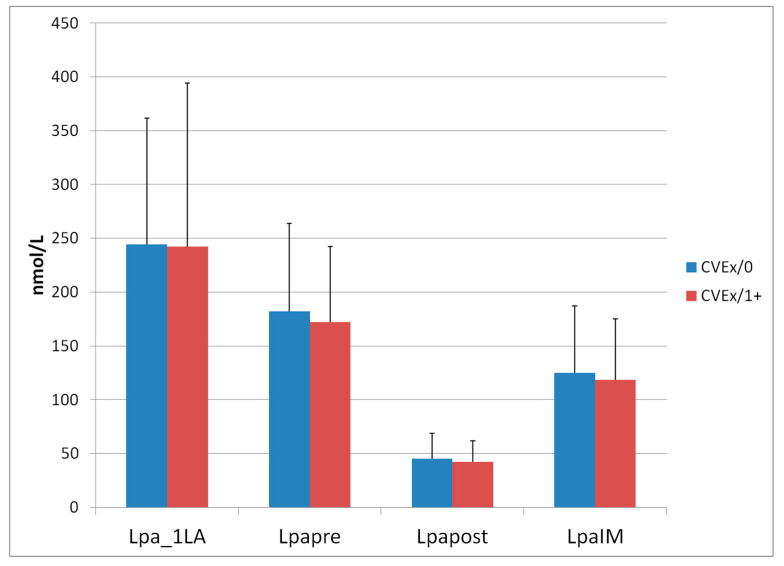
Lp(a) levels at first LA session and at an LA session (means, standard deviations) in 2019 (before (pre) and after (post) one single LA session) Patients with absent or low Lp(a) levels (Lp(a) was not measured in the clinical routine any longer): Group CVEx/0: 2, Group CVEx/1+: 10.

The LA therapy was very effective with respect to acute reductions of LDL-C. Lp(a) levels were also measured at the initiation of the LA therapy and at the same session as mentioned above in 2019 (Figure 3).

In contrast to the situation with LDL-C, a longer lasting LA therapy decreases the Lp(a) concentrations assessed before the LA sessions. Acute reductions of Lp(a) were also excellent.

In Table 2, the initial HDL-C and TG concentrations are given. Both parameters are influenced by LA but they do not represent target levels for the extracorporeal therapy. That is why no follow-up data are reported.

Clearly, some patients had a combined hyperlipoproteinemia with elevations of TG as well. The HDL-C values were low in some patients, in others extremely high. In the ANOVA testing, no difference was shown between the two CVE-Groups.

### 3.3. LA Methods Used

As mentioned in the methods section, six LA methods have been used (Table 3).

**Figure 4:**
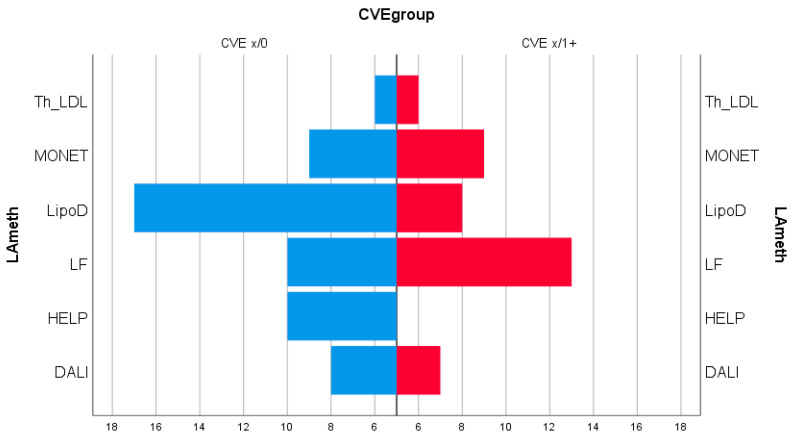
Number of patients treated with an LA method in the two groups CVEx/0 and CVEx/1+ (Abbreviations: Th_LDL TheraSorb^TM^LDL, LipoD Liposorber D, LF lipid filtration).

Age at start of the LA therapy, LDL-Cpre, LDL-CIM, Lp(a)pre, Lp(a)post, CVEsbef, and CVEsdur were not different between the LA methods subgroups. Figure 4 provides an information about the distribution of the LA methods among the two CVE-Groups.

The small numbers of patients in the subgroups of LA methods do not allow any final conclusions with respect to the effect of different LA methods on outcome data.

## 4. Discussion

The major findings in this investigation are that an older age at the start of the LA therapy and a higher number of CVEs in the history are associated with the incidence of CVEs during the extracorporeal treatment.

LDL-C and Lp(a) concentrations either measured initially nor before and after the LA sessions did not seem to have an influence. Of course, we were trying to optimize the acute reduction rates of both lipid fractions by modifying the treated volume of whole blood or plasma, by switching the patient to another LA method, and/or by optimizing the lipid-lowering drug therapy. In both groups the number of patients taking a statin was slightly decreased during the follow-up (most probably because of tolerance problems), whereas PCSK9 inhibitors were newly started in some patients, especially in Group CVEx/1+.

In the Pro(a)Life study when comparing with our data, patients suffered from a lower number of CVEs before the extracorporeal therapy, their mean age at the start of the LA therapy was 57 years (that is in the mean between the mean ages of our CVE-Groups). All CVEs were reduced by 75.9% when comparing the time periods 2 years before and during LA therapy. [3] The somewhat lower reduction rate of CVEs in our study may be explained by the longer observation time and the somewhat higher mean age in Group CVEx/1+.

In 2013, we had published a study in 64 apheresis patients, 20 of them developed new CVEs during LA therapy. [7] Among those with events there were relatively more males and relatively more old patients, the percentage of patients with elevated Lp(a) levels was higher, patients had suffered from more peripheral arterial occlusions and (re-)stenosis, and carotid artery stenosis (whereas other CVEs were not different from the other group without events). Mean lipid concentrations before and after LA were not different with the exception of higher LDL-Cpre levels in those patients who did not have new CVEs.

In the 2019 ESC/EAS Guidelines, a target LDL-C lower than 1.4 mmol/L (55 mg/dL) has been recommended for high risk patients. [12] The actual LDL-CIM values reflect the lipid load of the patients when treated extracorporeally. In Group CVEx/0 only 12 patients (20%) exhibited an LDL-CIM below 1.4 mmol/, in 28 patients (46.7%) the LDL-CIMcorr was below this level. The corresponding percentages for Group CVEx/1+ were: 20 patients (41.7%) (LDL-CIM) below 1.4 mmol/L and 31 patients (64.6%) (LDL-CIMcorr).

On the other hand, even rather low LDL-C concentrations do not guarantee an event-free future. This explains why in this guideline the authors recommend for patients with atherosclerotic vascular disease who experience a second vascular event within 2 years (not necessarily of the same type as the first event) while taking maximally tolerated statin-based therapy, an LDL-C goal of < 1.0 mmol/L (<40 mg/dL). The latter are patients in need for an LA therapy. Our data clearly point to the fact that the latter target can hardly be reached even in maximally treated patients. In Group CVEx/0 2 patients (3.3%) did not exceed this target (LDL-CIM) and 12 patients (25%) (LDL-CIMcorr), respectively. The data for Group CVEx/1+ were: 9 patients (18.8%) (LDL-CIM) and 17 patients (35.4%) (LDLCIMcorr). The data show that in Group CVEx/1+ the percentages were even higher than in the Group without new events during LA (CVEx/0).

For Lp(a) no therapeutic target values have been officially recommended up to now. In actuality, 120 (or higher) nmol/L is the officially accepted Lp(a) level for the indication to start an LA therapy in Germany [6]. In Group CVEx/0 the Lp(a)IM value was below 120 nmol/L (is equal to about 50 mg/dL) in 30 patients (50%), in Group CVEx/1+ in 21 patients (43.8%). The National Lipid Association defined a concentration of more than 100 nmol/L of Lp(a) as a risk-enhancing factor [13]. The mean Lp(a)IM values in both groups of this evaluation were higher than 100 nmol/L. When comparing the Lp(a) levels at the start of the LA therapy with the Lp(a)IM concentrations, in both groups a reduction by about 120 nmol/L was observed. Evidently, this reduction does not explain the difference in the occurrence of CVEs in these groups. It is not possible to interpret data obtained in Mendelian randomization analysis [13] with respect to lowering Lp(a) and its impact on cardiovascular outcome data from the point of view of the extracorporeal treatment.

LA methods may be different with respect to their pleiotropic effects [11]. The clinical relevance of these effects is still quite unclear. In the prospective Pro(a)Life Study, no difference with respect to outcome data has been detected when considering the various LA methods used. The differing distribution of LA methods in the two CVEs Groups in our evaluation which is depicted in Figure 4 evokes the suspicion that there could be some effects with respect to the occurrence of new events. However, the numbers in the subgroups are still rather small, and moreover the retrospective character of our study limits the significance of conclusions in this respect.

## 5. Conclusions

An LA therapy should be started earlier—already after 2 CVEs (or even only one) have occurred. The probability of new events will be higher when more than 3 CVEs have happened. Younger patients will have a higher benefit from the extracorporeal therapy. In the older patients, the atherosclerotic lesions may be more pronounced and may be more prone to develop complications.

The topic of this manuscript remains to be actual—future studies should include more patients (and could be done within the framework of the German Lipoprotein Apheresis Registry, where starting from January 2020 the LA methods have to be documented) [14]. Data looking at different apo(a) isoforms or genetic findings could be of interest.

## Figures and Tables

**Table 1:** LA methods used at our center.

LA Process	LA Method	Provider
Precipitation	HELP (heparin-induced extracorporeal LDL precipitation)	B. Braun Melsungen AG, Melsungen, Germany
Filtration	Lipid filtration	LF; DIAMED Medizintechnik GmbH, Cologne, Germany
	MONET (membrane filtration optimized novel extracorporeal treatment)	Fresenius Medical Care GmbH, Bad Homburg, Germany
Adsorption	DALI (direct adsorption of lipoproteins)	Fresenius Medical Care GmbH, Bad Homburg, Germany
	Liposorber D	Kaneka Pharma Europe N.V., Eschborn, Germany
	TheraSorb^TM^LDL	Antibodies; Miltenyi Biotec GmbH, Bergisch Gladbach, Germany

**Table 2:** HDL-C and TG levels (means, standard deviations, range; mmol/L) measured before the first LA session in both groups.

	CVEx/0	CVEx/1+
HDL-C_1LA	1.44 ± 0.36	1.42 ± 0.54
range	0.72–2.42	0.34–3.71
TG_1LA	1.98 ± 2.17	1.93 ± 1.26
range	0.42–15.63	0.56–5.74

**Table 3:** Numbers of patients treated with an LA method in May 2019. Parameters which were significantly different are listed: duration of LA therapy (mean, range), LDL-Cpost (after the LA session in 2019; mean ± standard deviation) LDL-Cpost: only the HELP and die Liposorber D groups were significantly different (Bonferroni-test: *p* 0.020).

LA Method	Numbers of Patients	Treatment (Years)	LDL-Cpost (mmol/L)
HELP	15	4.67 (range: 1.4–9.32)	0.93 ± 0.53
Lipid filtration	23	5.33 (range: 1.48–16.7)	0.69 ± 0.31
MONET	18	4.84 (range: 1.41–10.99)	0.64 ± 0.32
DALI	15	8.81 (range: 1.25–26.24)	0.71 ± 0.38
Liposorber D	25	5.68 (range: 1.67–11.09)	0.56 ± 0.25
TheraSorb^TM^LDL	12	4.46 (range: 1.41–14.73)	0.54 ± 0.24
